# Gastrointestinal Motility and Gut Hormone Secretion in response to Shenhuang Plaster in a Postoperative Ileus Rat Model

**DOI:** 10.1155/2021/8859579

**Published:** 2021-06-01

**Authors:** Yanan Shi, Yingsong Zheng, Jingming Xu, Bin Ding, Qiyang Shou, Guiping Chen, Ting Liu, Qiuhua Sun, Xiaohong Xu

**Affiliations:** ^1^School of Nursing, Zhejiang Chinese Medical University, Hangzhou 310053, China; ^2^The First Clinical Medical College, Zhejiang Chinese Medical University, Hangzhou 310053, China; ^3^College of Life Science, Zhejiang Chinese Medical University, Hangzhou 310053, China; ^4^School of Second Clinical Medical, Zhejiang Chinese Medical University, Hangzhou 310053, China; ^5^The First Affiliated Hospital of Zhejiang Chinese Medical University, Hangzhou 310018, China

## Abstract

Postoperative ileus (POI), a gastrointestinal function disorder, is a complication that arises from surgery. Shenhuang plaster (SHP) application to the Shenque acupoint (CV8) to promote the recovery of gastrointestinal function has achieved definite curative effects in clinical settings; however, the underlying pharmacological mechanism remains unknown. In this study, we evaluated the effects of SHP using a Sprague Dawley rat POI model. Then, gastrointestinal transit in different rat groups was evaluated by the movement of fluorescein-labelled dextran. Ghrelin, obestatin, motilin (MTL), and vasoactive intestinal peptide (VIP) plasma concentrations were measured via a radioimmunoassay. The expression of the ghrelin and obestatin receptors (GHS-R1*α* and GPR39) in the intestinal muscularis of rats in different groups was comparatively identified via western blotting. The results indicated that SHP application improved gastrointestinal motility in POI model rats. SHP application significantly increased ghrelin concentration and the expression of its receptor and inhibited obestatin concentration and the expression of its receptor in blood. Further, ghrelin concentration and the capability of gastrointestinal transit were positively correlated. Simultaneously, SHP application also promoted the secretion of other gastrointestinal motility hormones, such as MTL and VIP. Hence, these results provide evidence that SHP can promote the recovery of gastrointestinal transmission in POI rat models through regulation of ghrelin and other intestinal hormones.

## 1. Introduction

Postoperative ileus (POI) is a complication arising from abdominal or even non-abdominal surgery, generally manifested by varying degrees of abdominal pain, bloating, nausea, vomiting, weakened or missing bowel sounds, and even anastomotic leakage and infection [1]. POI is often considered the main reason for prolonged hospitalization and increased hospitalization costs [2], but its pathogenesis remains unknown. The influence of ghrelin on gastrointestinal motility has been investigated, and results from previous studies have indicated that the expression of ghrelin and its receptor (GHS-R1a, growth hormone secretagogue receptor) are repressed in POI [3]. This suggested that ghrelin may be a key regulator in POI.

In recent times, few effective treatments and therapies have been clinically useful in treating POI, including gastrointestinal decompression, anti-inflammatory rehydration, and nutritional supplementation [4]. However, the application of traditional Chinese medicine (TCM) external therapy presents advantages, such as exact curative effect, safe and easy application, and good patient compliance. For example, TCM plaster application on acupoints is a therapeutic method that has been practiced for over a thousand years [5, 6], and the effectiveness of acupuncture practices on gastrointestinal motility improvement has been well documented [7, 8].

POI is a kind of “intestinal knot.” Based on TCM theory, it is the obstruction of “Qi” (energy) in the intestinal tract, or the dysfunction of “Qi” [9, 10]. The Shenhuang plaster (SHP) is a “Qi”-promoting herbal formula consisting of Renshen (*Ginseng Radix Et Rhizoma*), Raw Dahuang (*Rhei Radix Et Rhizoma*), Danshen (*Salviae Miltiorrhizae Radix et Rhizoma*), Zhishi (*Aurantii Fructus Immaturus*), Houpo (*Magnoliae Officinalis Cortex*), Dingxiang (*Caryophylli Flos*), and Wuzhuyu (*Evodiae Fructus*) [11, 12]. The Shenque acupoint, CV8, is located in the “Ren” meridian, and it is the intersection point of the “Ren,” “Du,” and “Sanjiao” meridians, according to TCM literature [13]. Therefore, a Chinese doctor has suggested that treatment at the Shenque point will stimulate the “Qi” circulating throughout the body [14]. SHP has been applied clinically for years, and in previous studies we demonstrated that SHP can improve POI in different model animals [15]. We hypothesized that SHP can influence the expression of ghrelin as well as other intestinal hormones. Therefore, in this study, we elucidate our hypothesis using a POI rat model.

## 2. Materials and Methods

### 2.1. Animals

Sixty Sprague Dawley (SD) rats (male, 260–310 g body weight) were obtained from Slack Laboratory Animals Co., Ltd (certificate number, SCXK 2013-0016; Shanghai, China). All rats were housed under specific-pathogen-free (SPF) conditions at a constant temperature (23 ± 2°C) and humidity (55 ± 10%) with standard rodent chow, water ad libitum, and a 12-hour light/dark cycle. All animal experiments were performed according to the “Regulations for the Care and Use of Laboratory Animals in Zhejiang Chinese Medical University,” published by the Zhejiang Chinese Medical University. The institutional animal care and use committee of Zhejiang Chinese Medical University approved the study protocol, with certificate number 11722.

### 2.2. Chemical and Biochemical Materials

Fluorescein isothiocyanate- (FITC-) labelled dextran (70 kDa) was purchased from Thermo Fisher Scientific (Massachusetts, USA). Ghrelin, obestatin, motilin (MTL), and vasoactive intestinal peptide (VIP) (rat) radioimmunoassay kits were purchased from Phoenix Pharmaceuticals (California, USA). G Protein-Coupled Receptor 39 (GPR39) antibodies were obtained from Abcam (Cambridgeshire, UK). GHS-R1a (F-16; goat anti-mouse) primary antibody and rabbit anti-alkaline phosphatase- (AP-) labelled secondary antibody were purchased from Santa Cruz Biotechnology, Inc. (Santa Cruz, USA). Isoflurane was purchased from Lunan Pharmaceutical Co., Ltd. (Linyi, China). The 0.9% sodium chloride injection was provided by Zhejiang Sapais Pharmaceutical (Jiaxing, China), and PBS phosphoric acid buffer was provided by Zhongshan Bio. Co., Ltd. (Beijing, China).

### 2.3. Equipment

The NanoDrop 3300 spectrophotometer (Thermo Fisher Scientific, Massachusetts, USA), SDS-PAGE electrophoresis system (BIO-RAD, California, USA), inverted fluorescence microscope (Olympus Corporation, Tokyo, Japan), Allegra x-15r large capacity centrifuge (Beckman Coulter, Inc., California, USA), and ultra-low temperature freezer (ChangHong MeiLing Co. Ltd., Hefei, China) were used in this study.

### 2.4. Preparation of Shenhuang Plaster

The SHP recipe is an herbal mixture of 300 g Renshen (*Ginseng Radix Et Rhizoma*), 300 g Raw Dahuang (*Rhei Radix Et Rhizoma*), 300 g Danshen (*Salviae Miltiorrhizae Radix Et Rhizoma*), 200 g Zhishi (*Aurantii Fructus Immaturus*), 250 g Houpo (*Magnoliae Officinalis Cortex*), 125 g Dingxiang (*Caryophylli Flos*), and 125 g Wuzhuyu (*Evodiae Fructus*). These herbs were purchased from Chinese Herbal Pieces Co., Ltd. of Zhejiang Chinese Medical University, and they were qualified according to the standards noted in “The Pharmacopoeia of the People's Republic of China, 2015.” The ingredient extracts and SHP were prepared by the Traditional Chinese Medicine Preparation Institute of Zhejiang Chinese Medical University, as previously described [11].

### 2.5. Rat Surgery

The SD rat POI model preparation has been previously described [16, 17]. Briefly, the animals were fasted for 24 h before the experiment, with free access to water. The abdominal cavity of an isoflurane-anesthetized rat was opened with surgical scissors under sterile conditions. A gauze soaked with normal saline was placed on both sides of the incision, and two cotton applications with normal saline were used to roll and wipe from one site of the small intestine (near the stomach end) to the other site (near the cecum). Then, the operation was carried out several times from top to bottom in the same manner. Congestion and oedematous tissue were more obvious after scrubbing. Finally, the abdominal cavity and incision were sutured via a double-layered closure. The time period of small intestine manipulation was maintained within 10–15 min, during which the movement of intestinal contents was observed.

### 2.6. Animal Experimental Design and Treatment

SD rats were randomly separated into four groups, consisting of the saline treatment group (Ctrl), SHP treatment group (Ctrl + SHP), surgery/saline treatment group (POI), and surgery/SHP treatment group (POI + SHP). SHP treatment indicates that rats were administered SHP at the Shenque (CV8) point immediately after surgery. The SHP or saline plaster was changed twice a day during the experiment.

### 2.7. Capability of Gastrointestinal Transit Evaluation

The gastrointestinal transit evaluation method was described previously [11]. The rats were fasting for 24 h before each measured time point: 6 h, 12 h, 24 h, 48 h, and 72 h. Then, 200 *μ*L of FITC-labelled dextran (6.25 mg/ml, 70 kDa) was injected into each rat through the gastric tube. After 30 min, the rats were sacrificed by anaesthesia, and the abdominal cavity was opened. The total gastrointestinal tract was divided into 15 segments: the stomach (Sto), small intestine SI1–SI10 (SI; 10 segments), cecum (Ce), and colon Co1–Co3 (Co; 3 segments). Each segment of the intestinal lumen was washed with saline. The intestinal contents were discharged by centrifugation at 12000 rpm for 15 min. The supernatant was collected, and absorbance was measured with a photometer at 494 nm. The percentage absorbance per segment was calculated using equation (1). The geometric centre (GC), which indicated the distribution of fluorescein in the gastrointestinal tract, was calculated using equation (2).

(1) $$\begin{array}{c} \text{Percentage absorbance per segment}=\frac{\text{absorbance per segment}}{\text{total absorbance}}\times 10\text{0,} \end{array}$$

(2) $$\begin{array}{c} \text{GC}=\sum \frac{\left(\text{percentage of absorbance per segment}\times \text{number of segments}\right)}{100}. \end{array}$$

### 2.8. Determination of Plasma Ghrelin and Obestatin Concentrations

Rat blood was collected 6 h after surgery, and the ghrelin and obestatin concentrations (pg/ml) in plasma were determined using a standard curve obtained via gamma radioimmunoassay and the non-equilibrium method. At 6, 12, 24, 48, and 72 h each, 2 ml of blood was collected from the peritoneal vein, anticoagulated, and centrifuged at 3000 rpm and 4°C for 15 min. Then, the supernatant was aspirated into an Eppendorf tube and frozen at −20°C for testing. The “I” radioactivity in the precipitate was determined using the ghrelin and obestatin rat ultra-sensitive RIA Kit according to manufacturer's instructions.

### 2.9. Quantification of MTL and VIP Hormones

At the end of the experiment (72 h), 4 ml of peritoneal venous blood was collected into a 15 ml sterile blue-cap blood tube and allowed to stand at room temperature (18–25°C) for 2 h. Subsequently, it was centrifuged at 1000 rpm for 45 min, and the supernatant (serum) was removed and stored at −80°C. The MTL and VIP concentrations were determined via gamma radioimmunoassay, as previously described.

### 2.10. Western Blot Analysis

The expression of ghrelin and obestatin receptors in the intestinal muscularis of the different rat groups was compared via western blotting. After 72 h, the SD rats were sacrificed, and the colon (2–3 cm) was washed with 4°C PBS solution. The muscle layer was immediately cut off using surgery scissors, homogenized, and sonicated with radioimmunoprecipitation assay (RIPA) lysis buffer at 4°C. After 20 min centrifugation at 13500 rpm at 4°C, the supernatant was pipetted into a clean 1.5 ml centrifugation tube, and the protein concentration was quantitatively evaluated via the Bradford method. The target proteins were separated using 8% SDS polyacrylamide gel electrophoresis (SDS-PAGE), and the proteins in the gel were electro-transferred to the polyvinylidene fluoride (PVDF) membrane at 65 V for 2 h. The transferred PVDF membrane was blocked with blocking buffer containing 5% skim milk at 26°C for 1 h and hybridized with specific antibodies at 4°C overnight. After washing twice with a tween washing buffer (PBST) for 1 h, the hybridized PVDF membrane was incubated with AP-labelled secondary antibody for another 1 h. Before the addition of colour developer (NBT/BCTP) assay solutions, excess secondary antibodies were rinsed off thrice with PBST buffer.

### 2.11. Statistics

Data for each group were expressed as means ± standard deviation (SD). The statistical software SPSS 22.0 was used for analysis. The mean comparison between groups was analysed using one-way analysis of variance (ANOVA). When the variance was uniform, the *t*-test was used. When the variance was not uniform, the calibration *t*-test and linear correlation analysis were used. *P* < 0.05 was considered statistically significant.

## 3. Results

### 3.1. SHP Promotes Gastrointestinal Motility of POI Model Rats

**Figure 1:**
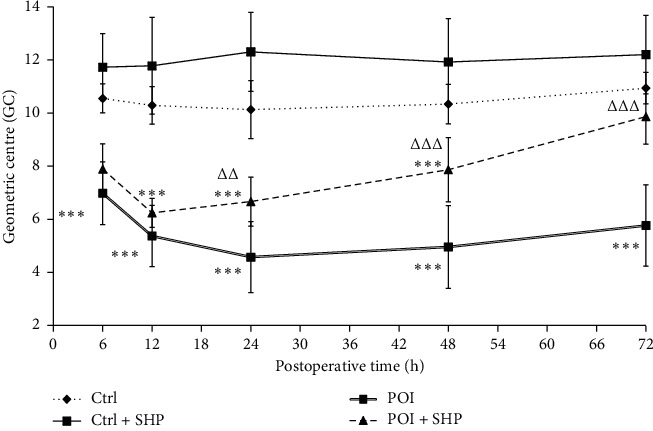
The dynamic change in the geometric centre of rats in different groups. The symbol ^∗∗∗^ indicates the statistical comparison with the Ctrl group (*P* < 0.001). The symbols △△ and △△△ indicate the statistical comparison with POI rats (*P* < 0.01 and *P* < 0.001, respectively). Ctrl, control; SHP, Shenhuang plaster; POI, postoperative ileus.

The FITC-labelled dextran GC in the intestine, which can intuitively indicate the speed of gastrointestinal motility, was used in this study to determine gastrointestinal motility. The GC value of the Ctrl group maintained a dynamic balance at each detection time (Figure 1). Meanwhile, the GC of the Ctrl + SHP group was greater than that of the Ctrl group at each time, indicating motility improvement by SHP; however, this was not statistically significant (*P* > 0.05). The GCs of the two surgery groups were significantly lower than those of the Ctrl group at 48 h (^∗∗∗^*P* < 0.001). At 72 h, the GC of the POI + SHP group recovered, but it was not significantly different from that of the Ctrl group. Hence, the GC of the POI group was significantly lower than Ctrl GC at 48 h (^∗∗∗^*P* < 0.001). The recoveries were also obtained. In detail, the GC values of the POI group were 6.98 ± 0.42, 5.37 ± 0.41, 4.57 ± 0.66, 4.95 ± 0.55, and 5.76 ± 0.54 at 6, 12, 24, 48, and 72 h, respectively. This shows a decrease within 24 h and a slow recovery after 24 h. The GC of the POI + SHP group decreased within 12 h and then recovered swiftly and significantly (ΔΔ*P* < 0.01, ΔΔΔ*P* < 0.001) compared to the POI group. The GC values of each group are listed in Table 1. This result indicated that SHP stimulated gastrointestinal motility.

### 3.2. SHP Stimulated the Expression of Ghrelin in Rats

**Figure 2:**
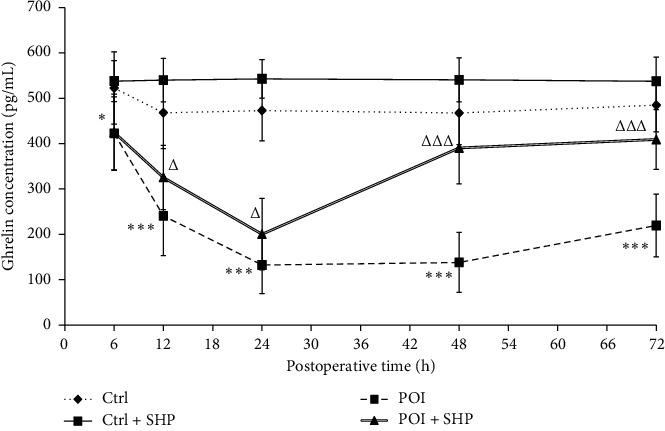
The concentration of ghrelin in different groups at different times. The symbols ^*∗*^ and ^∗∗∗^ indicate the statistical comparison with the Ctrl group (*P* < 0.05 and *P* < 0.001, respectively). The symbols △ and △△△ indicate the statistical comparison with POI (*P* < 0.05 and *P* < 0.001, respectively). Ctrl, control; SHP, Shenhuang plaster; POI, postoperative ileus.

**Figure 3:**
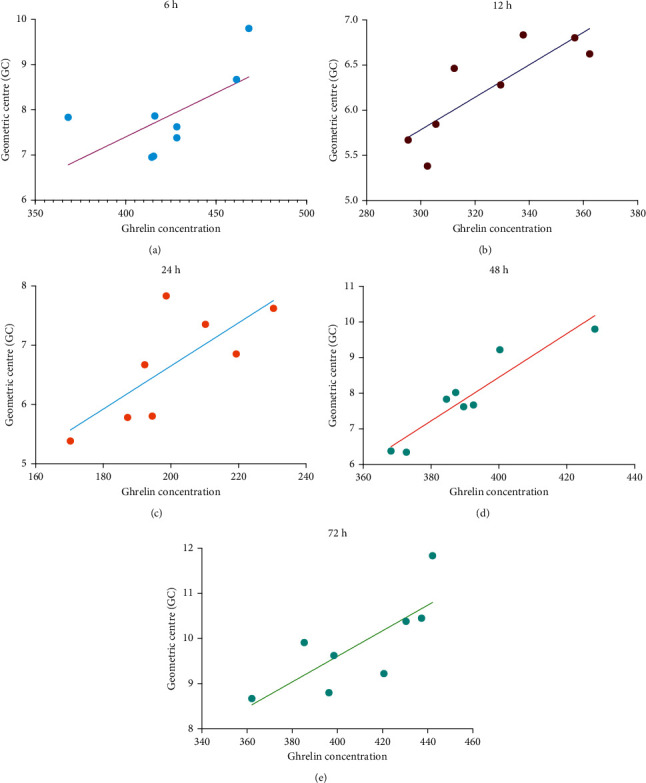
The correlation of changes between the ghrelin concentration and gastrointestinal transit centre of rats in the POI + SHP group. SHP, Shenhuang plaster; POI, postoperative ileus.

After surgery, the ghrelin concentration in blood changed dynamically in the different groups (Figure 2). Ghrelin concentrations in the Ctrl and Ctrl + SHP groups were maintained between 490 and 520 pg/ml from 12 to 72 h, respectively. However, no significant difference was observed between both groups (*P* < 0.05). Compared with the Ctrl group, the ghrelin concentration in the POI group decreased significantly, especially between 24 h (132.24 ± 22.20) and 48 h (138.06 ± 23.38). However, the ghrelin concentrations in rats in the POI + SHP group at 6, 12, 48, and 72 h were 425.03 ± 29.76, 325.28 ± 25.00, 390.43 ± 28.10 (ΔΔΔ*P* < 0.001, with the POI group), and 409.11 ± 23.29 pg/ml, respectively. The ghrelin concentration significantly recovered within 48 h. Hence, it remained lower than that of rats in the Ctrl group, but not significantly. Correlation analyses between ghrelin concentration and the GC of the gastrointestinal transit of rats in the POI + SHP group are shown in Figure 3, and the correlation coefficients for both physiological indices were 0.633, 0.836, 0.898, 0.935, 0.768, and 0.547 at 6, 12, 24, 48, and 72 h, respectively.

### 3.3. Dynamic Changes in Obestatin Concentrations among Different Groups

**Figure 4:**
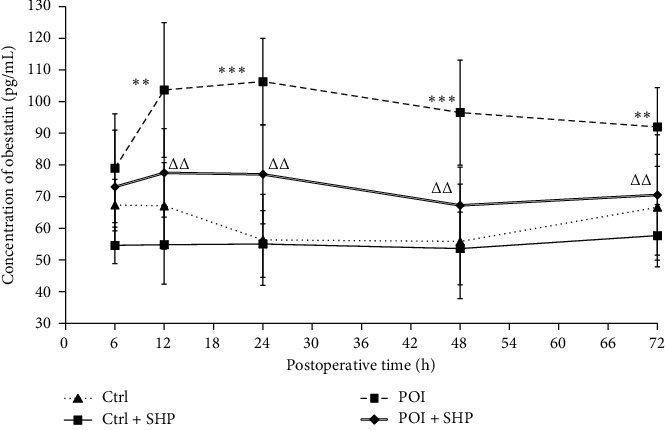
The concentration of obestatin in different groups at different times. The symbols ^∗∗^ and ^∗∗∗^ indicate the statistical comparison with the Ctrl group (*P* < 0.01and *P* < 0.001, respectively). The symbol △△indicates the statistical comparison with POI (*P* < 0.01). Ctrl, control; SHP, Shenhuang plaster; POI, postoperative ileus.

The obestatin concentrations in rats in different groups are listed in Table 2. No significant changes were observed in the two groups (Ctrl and Ctrl + SHP) of rats that underwent placebo surgery. Instead, the obestatin levels in rats in the POI group increased significantly at 12 h after surgery. Additionally, SHP administration attenuated obestatin expression in the POI group within the same period (12 h; Figure 4).

### 3.4. SHP Influenced the Expression of GHS-R1*α* and GPR39 in Smooth Muscle Cells of the Jejunum in Different Groups

**Figure 5:**
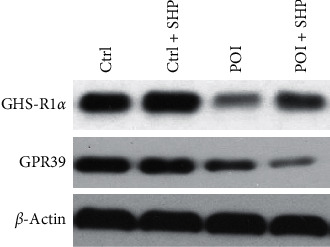
The expression of GHS-R1*α* and GPR39 in the colon muscularis of rats from different groups at 72 h after operation. Ctrl, control; SHP, Shenhuang plaster; POI, postoperative ileus.

GHS-R1*α* and GPR39 are the respective receptors for ghrelin and obestatin in intestinal smooth muscle cells. The effect of SHP on the expression of GHS-R1*α* and GPR39 was investigated via western blotting (Figure 5). Compared to the Ctrl group, GHS-R1*α* expression in the POI group was significantly repressed, and the effect of SHP treatment on GHS-R1*α* expression in the Ctrl + SHP and POI + SHP groups was evident. GPR39 expression was identical in the Ctrl and Ctrl + SHP groups but attenuated in the POI and POI + SHP groups.

### 3.5. Serum MTL and VIP Level of Rats Were Improved by SHP

**Figure 6:**
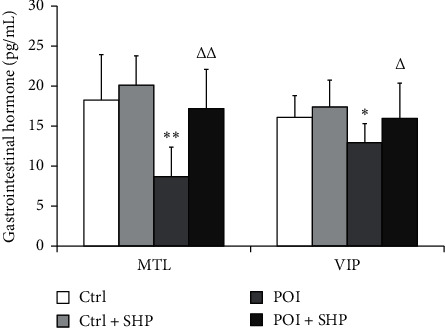
The transcriptional expression of MTL and VIP in rats from different groups at 72 h after operation. The symbols ^*∗*^, ^∗∗^, and ^∗∗∗^ indicate the statistical comparison with the Ctrl group, *P* < 0.05, *P* < 0.01, and *P* < 0.001, respectively. The symbols △ and △△ indicate the statistical comparison with POI, *P* < 0.05 and *P* < 0.01, respectively. MTL, motilin, MTL; VIP, vasoactive intestinal peptide; Ctrl, control; SHP, Shenhuang plaster; POI, postoperative ileus.

To elucidate the effects of SHP on gastrointestinal motility, the transcription of MTL and VIP was semi-quantified using ELISA. Compared to the Ctrl group, the transcription of both gastrointestinal hormones in the Ctrl + SHP group increased, but not significantly (Figure 6). After surgery, the transcription of MTL and VIP was significantly diminished (^*∗*^*P* < 0.05, ^∗∗^*P* < 0.01, ^∗∗∗^*P* < 0.001), and SHP administration improved the effects of surgery (Δ*P* < 0.05, ΔΔ*P* < 0.01).

## 4. Discussion

The pathogenesis of POI is complicated, and it is generally considered to be controlled by multiple factors, such as perioperative medication, gastrointestinal hormone changes, water-electrolyte disorders, and surgical trauma. One such major pathological change is small intestinal smooth muscle inflammation [1]. Currently, the aetiology and pathogenesis of POI remain unclear, and clinical treatment mainly relies on rapid rehabilitation concepts for comprehensive treatment, such as reducing surgical trauma, limiting fluid replacement, and early restoration of diet and activities, but the clinical effects of these therapies are not satisfactory [18]. The TCM treatment of POI is not unique, as POI is considered a syndrome that combines a deficiency and reality in TCM, which is classified as “intestinal obstruction.” The pathogenesis of POI is mainly due to the suffocation of traumatic “Qi” and blood consumption. Moreover, a “Qi” deficiency impedes blood circulation [19, 20]. Therefore, improving “Qi” in the intestine could improve the symptoms of POI. Moreover, the prescription of SHP has complied with the TCM aetiology and pathogenesis theory of promoting the motility of “Qi.” In the last decade, SHP application at the “Shenque” point has been clinically beneficial for POI and constipation treatment (unpublished clinical trial results), but the therapeutic mechanism remains unknown. SHP consists of seven herbs, which contain >100 chemical ingredients. Therefore, the mechanism of action is difficult to elucidate, due to its “multi-component, multi-target, and multi-pathway” properties. It has been reported that several active ingredients in TCMs have anti-inflammatory and gastrointestinal motility effects that are compatible with SHP [21–23]. Our previous studies showed that SHP enhanced gastric motility and could potentially serve as a novel therapeutic agent for chemotherapeutic constipation due to its anti-inflammatory potency [11].

POI is a common clinical complication following colon/rectal surgery [1]. Generally, small bowel peristalsis is restored 24 h after surgery, gastric peristalsis at 24 to 48 h, and colonic peristalsis between 48 and 72 h [24]. However, the recovery of gastrointestinal function in patients with POI is even longer. Serious complications, such as intestinal flora shift and multiple organ dysfunction syndrome, were reported in some cases [25]. In the current study, the GC was significantly larger in the POI group than in the Ctrl group at 72 h after surgery (Figure 1 and Table 1), indicating that this method was appropriate for POI rat model preparation.

In this study, the recovery of gastrointestinal transit (determined as GC) in the POI + SHP group was observed at 24 h. The recovery of ghrelin concentration in the serum was at 48 h (Figure 2), and the GC values were positively correlated with ghrelin concentrations in the POI + SHP group (Figure 3). On the contrary, the concentration of obestatin in the POI and POI + SHP groups increased after surgery. However, a significant difference was observed in the POI + SHP group (vs. POI group) 12 h after surgery (Figure 4). Additionally, the regulative activity of SHP on GHS-R1*α* [26] and GPR39 [27, 28] was identified in different groups via western blotting (Figure 5). The transcription of MTL and VIP in different groups was detected, and the influence of SHP on both hormones was elucidated (Figure 6).

Abnormal defecation is a pathological feature in many diseases and a major symptom in TCM diagnoses. Recent medical studies indicated that the enteric nervous system, smooth muscle, and interstitial cells are involved in gastrointestinal motility regulation [29, 30]. Some functional molecules have been discovered, such as protein hormones, ghrelin, obestatin [31], MTL [32], and VIP [33], which serve as key targets for the development and evaluation of novel therapies.

Ghrelin, an endogenous brain-gut peptide, first discovered by the Japanese scholar Kojima in 1999 in the stomach of rats, can directly promote gastrointestinal motility by binding to GHS-Rla [26, 34]. In this study, intestinal damage (in the POI group, but not in the Ctrl group) attenuated ghrelin expression from 6 h to 24 h after operation, in accordance with a previous study [35]. Then, the ghrelin concentration remained at a low level within 72 h. Compared to the POI group, the serum ghrelin level in the POI + SHP group increased significantly 24 h after surgery. However, there was no significant difference between the Ctrl and Ctrl + SHP groups (Figure 2), suggesting that SHP promoted gastrointestinal motility by improving ghrelin secretion in rats with damaged intestines, but not in healthy ones. A similar effect of SHP on GHS-Rla in the colon muscularis of rats from different groups was elucidated (Figure 5).

Obestatin, an active polypeptide in the blood circulation, manifests several biological functions, such as inhibition of thirst [36] and food intake [37, 38], improvement of memory retention [39], regulation of sleep quantity and quality [40], and gastrointestinal motility attenuation [31]. Obestatin, mainly secreted by the intestine, can control gastric emptying speed and reduce intestinal contraction [41], which means that obestatin inhibits intestinal motility. Our results indicated the inhibitory activity of SHP in POI-induced obestatin (Figure 4) and GPR39 (Figure 5) overexpression. The results suggested that SHP application regulated ghrelin and obestatin expression simultaneously, possibly as a result of the similarity in ghrelin and obestatin gene codes [42].

MTL and VIP, which exist in several animals, are gastrointestinal motility-stimulating hormones. Hence, previous reports have noted an interaction between motilin and ghrelin in regulating GI motility [43]. Moreover, these two hormone peptides and their respective regulators share partial identities and structures [44], and this study showed that both hormones were influenced by surgery and SHP application (Figures 2 and 6). However, in humans, endogenous levels of ghrelin were not correlated with motilin levels [45, 46]. Therefore, we speculate that ghrelin and motilin have their own unique functions, apart from their similar activities in promoting gastrointestinal motility.

VIP, present in different organs, has been recognized as a neuropeptide [47], which can mediate several gastrointestinal functions, such as gastric acid and intestinal anion secretion, enzyme release from the pancreas, cellular motility, vasodilation, and intestinal contractility [48–50]. However, the function of its receptors, VPAC1 and VPAC2, in gastrointestinal motility stimulation remains unclear [33].

Meanwhile, the use of SHP as an external POI treatment avoids intravenous infusion and transgastric administration, which is especially beneficial for patients who need to limit fluid volume and undergo gastrointestinal surgery. SHP is safe to use and is also simple, inexpensive, and easy; however, further studies are required to evaluate this therapeutic approach.

## 5. Conclusions

SHP application at the “Shenque” acupoint is an effective and safe therapeutic method for POI amelioration. However, the pharmacological mechanism remains unknown because of its complicated ingredients and external application. In this study, we first demonstrated the gastrointestinal motility-promoting capability of SHP administered in a POI rat model. Following this, the influence of SHP application on the expression of ghrelin, obestatin, and their receptors in the colon muscularis was demonstrated. Further, improvements in SHP effects based on the concentration of the serum gastrointestinal hormones MTL and VIP were identified in POI rats, but elucidating the underlying mechanism of action requires further study.

## Figures and Tables

**Table 1:** The GC values for each group.

Group	6 h	12 h	24 h	48 h	72 h
Ctrl	10.55 ± 1.68	10.29 ± 1.06	10.13 ± 1.09	10.34 ± 1.09	10.94 ± 1.04
Ctrl + SHP	11.73 ± 1.26	11.78 ± 1.82	12.30 ± 1.49	11.92 ± 1.63	12.20 ± 1.48
POI	6.98 ± 1.19	5.37 ± 1.15	4.57 ± 1.34	4.95 ± 1.56	5.76 ± 1.53
POI + SHP	7.89 ± 0.95	6.24 ± 0.55	6.66 ± 0.92	7.86 ± 1.21	9.86 ± 1.04

GC, geometric centre; Ctrl, control; SHP, Shenhuang plaster; POI, postoperative ileus.

**Table 2:** The obestatin concentration in each group at different time points.

Group	6 h	12 h	24 h	48 h	72 h
Ctrl	67.32 ± 8.16	67.09 ± 13.67	56.34 ± 14.37	55.84 ± 18.11	66.64 ± 16.68
Ctrl + SHP	54.59 ± 5.77	54.83 ± 12.46	55.04 ± 10.52	53.59 ± 11.51	57.67 ± 9.82
POI	78.97 ± 17.15	103.67 ± 21.27	106.32 ± 13.67	96.54 ± 16.55	91.98 ± 12.42
POI + SHP	73.06 ± 17.93	77.50 ± 13.96	77.03 ± 15.66	67.23 ± 12.10	70.54 ± 19.00

Note: the significant difference is noted in Figure 4. Ctrl, control; SHP, Shenhuang plaster; POI, postoperative ileus.

## Data Availability

All data used to support the findings of this study are included within the article, and these data can also be accessible on website https://fairsharing.org/collection/GastrointestinalMotilityandGutHormoneSecretioninResponsetoShenhuangPlasterinaRatModelofPostoperativeIleus.
